# Stromal p16 expression is significantly increased in endometrial carcinoma

**DOI:** 10.18632/oncotarget.13594

**Published:** 2016-11-25

**Authors:** Gun Yoon, Chang Won Koh, Nara Yoon, Ji-Ye Kim, Hyun-Soo Kim

**Affiliations:** ^1^ Shinsegae Women's Hospital, Daegu, Republic of Korea; ^2^ Department of Obstetrics and Gynecology, Seoul National University Hospital, Seoul, Republic of Korea; ^3^ Department of Pathology, The Catholic University of Korea Incheon St. Mary's Hospital, Incheon, Republic of Korea; ^4^ Department of Pathology, Severance Hospital, Yonsei University College of Medicine, Seoul, Republic of Korea

**Keywords:** p16, endometrium, peritumoral stroma, endometrioid carcinoma, serous carcinoma

## Abstract

p16 is a negative regulator of cell proliferation and is considered a tumor suppressor protein. Alterations in p16 protein expression are associated with tumor development and progression. However, the p16 expression status in the peritumoral stroma has not been investigated in the endometrium. Therefore, we evaluated stromal p16 expression in different types of endometrial lesions using immunohistochemistry. Differences in the p16 expression status according to the degree of malignancy and histological type were analyzed. This study included 62, 26, and 36 cases of benign, precancerous, and malignant endometrial lesions, respectively. Most benign lesions showed negative or weak expression, whereas precancerous lesions showed a variable degree of staining proportion and intensity. Atypical hyperplasia/endometrial intraepithelial neoplasia (AH/EIN) and serous endometrial intraepithelial carcinoma (SEIC) had significantly higher stromal p16 expression levels than benign lesions. Endometrioid carcinoma (EC), serous carcinoma (SC), and carcinosarcoma showed significantly elevated stromal p16 expression levels compared with benign and precancerous lesions. In addition, there were significant differences in stromal p16 expression between AH/EIN and SEIC and between EC and SC. In contrast, differences in stromal p16 expression among nonpathological endometrium, atrophic endometrium, endometrial polyp, and hyperplasia without atypia were not statistically significant. Our observations suggest that stromal p16 expression is involved in the development and progression of endometrial carcinoma, and raise the possibility that p16 overexpression in the peritumoral stroma is associated with aggressive oncogenic behavior of endometrial SC.

## INTRODUCTION

Endometrial carcinoma is the commonest pelvic gynecological cancer in the westernized parts of the world [1, 2]. Worldwide, the incidence of endometrial carcinoma is rapidly increasing, with the highest disease burden reported in North America and Western Europe. In the United States, if current trends continue, then there will be a doubling in the number of women diagnosed with endometrial carcinoma by the year 2030 to 122,000 cases per year [1, 3]. These trends have extended globally. In particular, Korea has witnessed a doubling of the age-standardized incidence of endometrial carcinoma [4, 5].

p16 is the principal member of the INK4 family of cyclin-dependent kinase (CDK) inhibitors [6]. As a regulatory protein of the cell cycle, p16 is involved in G1-to-S phase transition. Upon binding to CDK4/6, p16 inhibits the cyclin D1-CDK4/6 complex formation and CDK4/6-mediated phosphorylation of the retinoblastoma (RB) protein. Once RB is phosphorylated, the E2F-RB complex dissociates, leading to reduced growth-suppressor activity of RB [7]. Like RB protein, p16 is a tumor suppressor. p16 maintains RB protein family members in a hypophosphorylated state [7–10]. However, it is difficult to explain many aspects of p16 function and regulation by its well-known function as a tumor suppressor alone. In addition, the molecular pathways responsible for p16 function and expression have not yet been elucidated.

p16 expression has been analyzed in some studies of gynecological malignancy. According to the 2014 World Health Organization (WHO) Classification, more than half of ovarian high-grade serous carcinomas show diffuse, strong p16 expression [11]. The majority of endometrial serous carcinoma (SC) also strongly expresses p16 protein. p16 and p53 expression levels are used as differential immunostaining markers to distinguish high-grade serous carcinoma from other histological types of ovarian carcinoma and SC from other histological types of endometrial carcinoma. In addition, p16 overexpression occurs in human papillomavirus (HPV)-related tumors [6, 11]. p16 overexpression indicates high-risk HPV infection, not only in uterine cervical squamous cell carcinoma and usual-type endocervical adenocarcinoma, but also in endocervical adenocarcinoma *in situ* and high-grade squamous intraepithelial lesions of the vulva, vagina, and anogenital regions. Therefore, p16 is used as a diagnostic biomarker for HPV-related precancerous lesions and invasive carcinoma [6, 12].

However, conflicting patterns of p16 expression have been reported, which further complicates the understanding of its biological and pathological roles. p16 expression is either lost or downregulated [13–16], or clearly overexpressed [17–21], in different types of malignancy. Recently, during the routine diagnosis of surgically resected or curetted endometrial neoplasms, we noticed p16 expression in the peritumoral stroma, as well as in the glandular epithelial cells. The levels of p16 expression in the stromal cells varied depending on the degree of malignancy and histological type of endometrial lesions. Although p16 is commonly used as a biomarker for diagnosing gynecological malignancies, its expression in the stromal component of endometrial neoplasms has seldom been studied. In this study, we examined stromal p16 expression in benign, precancerous, and malignant endometrial neoplasms using immunohistochemical staining and investigated whether a significant difference exists in stromal p16 immunoreactivity according to the degree of malignancy and/or histological type.

## RESULTS

### Patient demographics

This preliminary study was conducted with 124 patients who underwent curettage, polypectomy, or hysterectomy for benign, precancerous, or malignant lesions of the endometrium from March 2015 to May 2016. Classification of all 124 cases according to the degree of malignancy of endometrial lesions resulted in 62 benign lesions, 26 precancerous lesions, and 36 malignant lesions. The age of the patients ranged from 25 to 74 years (median, 46 years) in patients with benign lesions, from 27 to 67 years (median, 51 years) in patients with precancerous lesions, and from 41 to 84 years (median, 59 years) in patients with malignant lesions. According to histological type, the 62 benign lesions consisted of nonpathological endometrium (10 cases), atrophic endometrium (13 cases), adenomyosis (8 cases), endometrial polyp (17 cases), and hyperplasia without atypia (14 cases). The 26 precancerous lesions consisted of atypical hyperplasia/endometrial intraepithelial neoplasia (AH/EIN; 21 cases) and serous endometrial intraepithelial carcinoma (SEIC; 5 cases). The 36 malignant lesions consisted of endometrioid carcinoma (EC; 21 cases), SC (8 cases), and carcinosarcoma (7 cases).

### Stromal p16 expression in benign, precancerous, and malignant endometrial lesions

**Figure 1:**
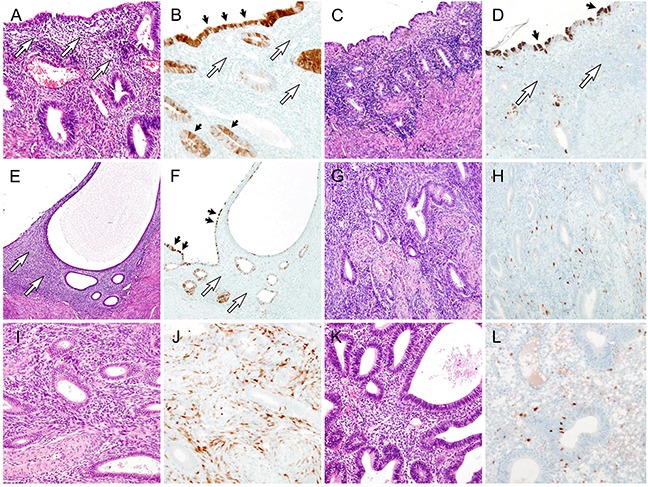
Stromal p16 expression in benign endometrial lesions **A**. Nonpathological endometrium. White arrows indicate endometrial stroma. **B**. The endometrial stromal cells (white arrows) do not exhibit p16 immunoreactivity, whereas the endometrial glandular epithelium (black arrows) displays patchy p16 expression, with variable staining intensities. **C**. Atrophic endometrium. **D**. The glandular epithelium (black arrows) exhibits scattered p16 immunoreactivity, whereas in the stroma (white arrows), p16 expression is absent. **E**. Adenomyosis. White arrows indicate endometrial stroma. **F**. This adenomyotic focus has an identical p16 expression pattern to that of nonpathological endometrium. None of the stromal cells (white arrows) reacts with p16 protein. Note scattered p16 immunoreactivity in the glandular epithelium (black arrows). **G**. Endometrial polyp. The presence of thick-walled blood vessels is a characteristic finding of endometrial polyp. **H**. A few scattered stromal cells exhibit weak to moderate p16 immunoreactivity. **I**. Endometrial polyp. **J**. In contrast with image H, approximately one-half of stromal cells display moderate p16 immunoreactivity. **K**. Hyperplasia without atypia. **L**. The stromal p16 expression level in hyperplasia without atypia is similar to that in endometrial polyp (image H).

The p16 immunostaining scores of benign, borderline, and malignant endometrial lesions are presented in Table 1. Representative photomicrographs of stromal p16 expression in benign endometrial lesions are presented in Figure 1. All 62 cases of benign endometrial neoplasm showed p16 immunostaining scores of ≤ 3. p16-positive stromal cells were randomly distributed throughout the entire endometrial thickness. There was no significant difference in stromal p16 expression between the surface and basal endometrium. Of the 62 cases of benign lesions, 33 cases (53.2%) showed no p16 expression, whereas 16 cases (25.8%), 10 cases (16.1%), and 3 cases (4.8%) had scores of 1, 2, and 3, respectively. Two (11.8%) and one (7.1%) cases of endometrial polyp and hyperplasia without atypia, respectively, exhibited scores of 3. No significant difference was observed in stromal p16 expression status among the histological types (*P* = 0.135). Of the 10 cases of nonpathological endometrium, two cases (20.0%) were found to have regional heterogeneity in stromal p16 expression. In both cases, approximately one-half of the stromal cells displayed weak p16 immunoreactivity (score 2). In the majority of benign endometrial lesions, the endometrial glandular epithelium displayed patchy, weak to moderate cytoplasmic p16 immunoreactivity. p16-positive endometrial glands were randomly distributed throughout the entire endometrium.

**Table 1:** Stromal p16 expression in benign, precancerous, and malignant endometrial lesions

Category	Pathological diagnosis	Total	p16 immunostaining score						
			0	1	2	3	4	6	9
Benign	Nonpathological endometrium	10	5 (50.0)	3 (50.0)	2 (50.0)	0 (0.0)	0 (0.0)	0 (0.0)	0 (0.0)
	Atrophic endometrium	13	9 (69.2)	3 (23.1)	1 (7.7)	0 (0.0)	0 (0.0)	0 (0.0)	0 (0.0)
	Adenomyosis	8	6 (75.0)	0 (0.0)	2 (25.0)	0 (0.0)	0 (0.0)	0 (0.0)	0 (0.0)
	Endometrial polyp	17	8 (47.1)	3 (17.6)	4 (23.5)	2 (11.8)	0 (0.0)	0 (0.0)	0 (0.0)
	Hyperplasia without atypia	14	5 (35.7)	7 (50.0)	1 (7.1)	1 (7.1)	0 (0.0)	0 (0.0)	0 (0.0)
Precancerous	AH/EIN	21	1 (4.8)	3 (14.3)	4 (19.0)	5 (23.8)	6 (28.6)	2 (9.5)	0 (0.0)
	SEIC	5	0 (0.0)	0 (0.0)	0 (0.0)	0 (0.0)	1 (20.0)	2 (40.0)	2 (40.0)
Malignant	Endometrioid carcinoma	21	2 (9.5)	0 (0.0)	1 (4.8)	4 (19.0)	4 (19.0)	7 (33.3)	3 (14.3)
	Serous carcinoma	8	0 (0.0)	0 (0.0)	0 (0.0)	0 (0.0)	1 (12.5)	3 (37.5)	4 (50.0)
	Carcinosarcoma	7	0 (0.0)	0 (0.0)	0 (0.0)	0 (0.0)	1 (14.3)	4 (57.1)	2 (28.6)

**Abbreviations:** AH/EIN: atypical hyperplasia/endometrioid intraepithelial neoplasia, SEIC: serous endometrial intraepithelial carcinoma

**Figure 2:**
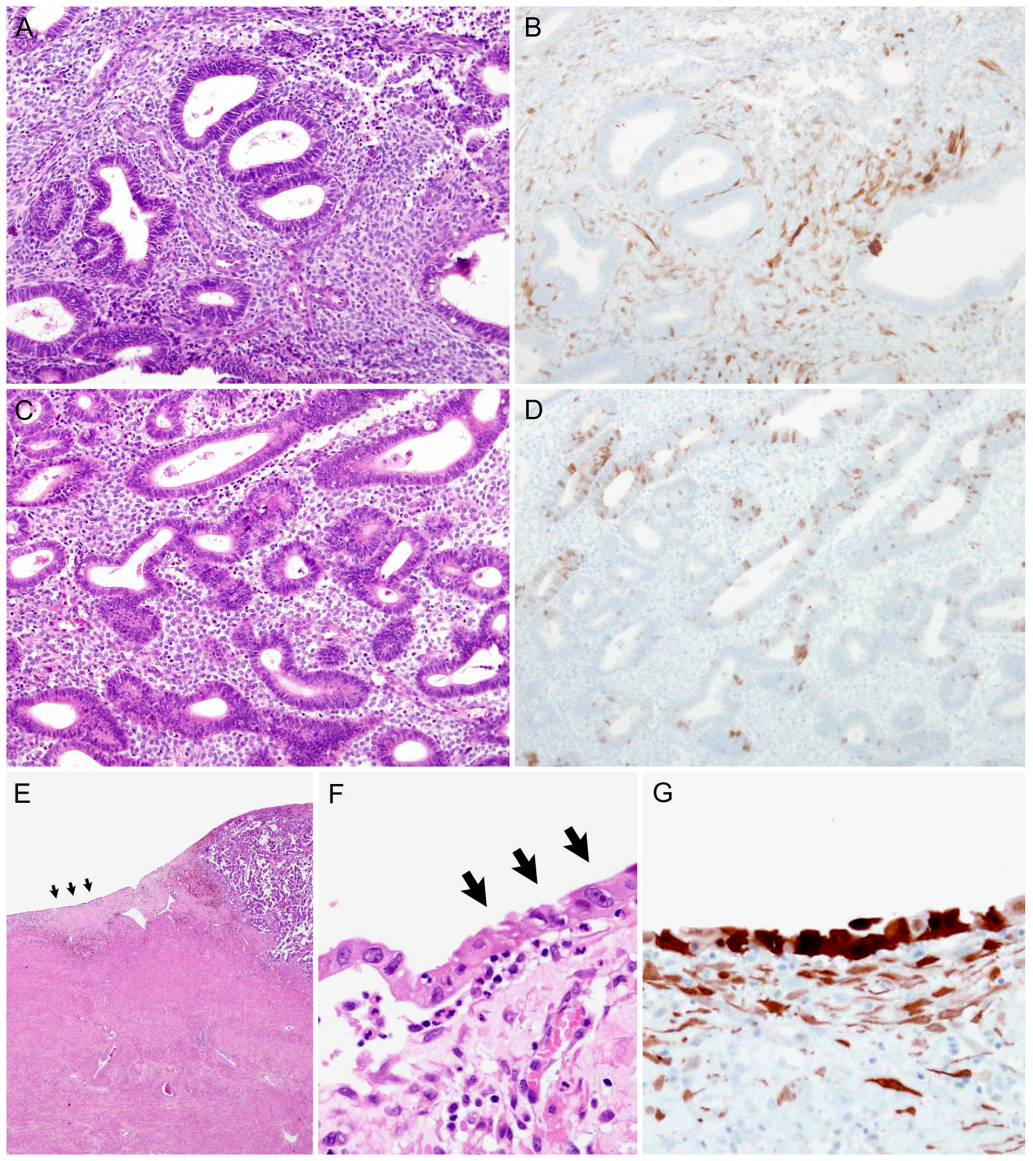
Stromal p16 overexpression in precancerous endometrial lesions: Atypical hyperplasia/endometrioid intraepithelial hyperplasia (AH/EIN) and serous endometrial intraepithelial carcinoma (SEIC) **A**. AH/EIN. **B**. A few scattered stromal cells exhibit weak to moderate p16 immunoreactivity. **C**. AH/EIN. **D**. None of the stromal cells reacts with p16 protein. Note scattered p16 immunoreactivity in the glandular epithelium. **E**. SEIC. Serous carcinoma (SC) is present at the right upper corner. Black arrows indicate SEIC, which locates adjacent to the SC. **F**. High-power view (×200) of SEIC (black arrows) displays a single layer of neoplastic epithelial cells showing severe nuclear pleomorphism. The stromal cells are irregularly distributed and admixed with inflammatory cells. **G**. The tumor cells strongly react with p16. The stromal cells also display diffuse, moderate p16 immunoreactivity.

Representative photomicrographs of stromal p16 expression in precancerous endometrial lesions are presented in Figure 2. Of the 26 cases of precancerous endometrial lesions, 18 (69.2%) showed p16 immunostaining scores of ≥ 3. Six (28.6%) and two (9.5%) cases of AH/EIN had scores of 4 and 6, respectively. While the scores of AH/EIN varied from 0 to 6, those of SEIC were ≥ 4. Four (80.0%) of the 5 SEIC cases showed scores of ≥ 6. In contrast with the benign lesions, there was a significant difference in stromal p16 expression status between AH/EIN and SEIC (*P* = 0.001); stromal p16 expression was significantly higher in SEIC than in AH/EIN. All cases of SEIC exhibited strong p16 staining intensity in almost all of the tumor cells, whereas none of the AH/EIN cells showed diffuse expression. Fifteen (71.4%) of the 21 AH/EIN cases exhibited patchy, weak to moderate cytoplasmic p16 immunoreactivity in the tumor cells.

**Figure 3:**
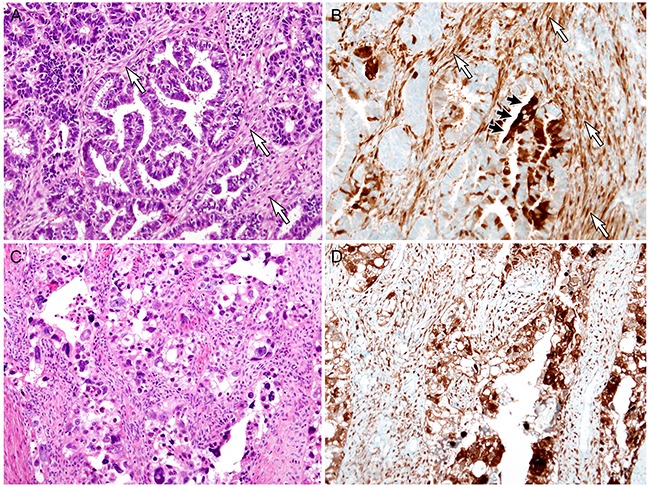
Stromal p16 overexpression in malignant endometrial lesions: Endometrioid carcinoma (EC) and serous carcinoma (SC) **A**. EC. White arrows indicate the periglandular stroma. **B**. p16 strongly highlights the spindle-shaped stromal cells (white arrows) that are distributed between neoplastic glands. The tumor cells (black arrows) show patchy p16 expression. **C**. SC. **D**. Both the tumor cells and stroma exhibit diffuse, strong p16 immunoreactivity.

**Figure 4:**
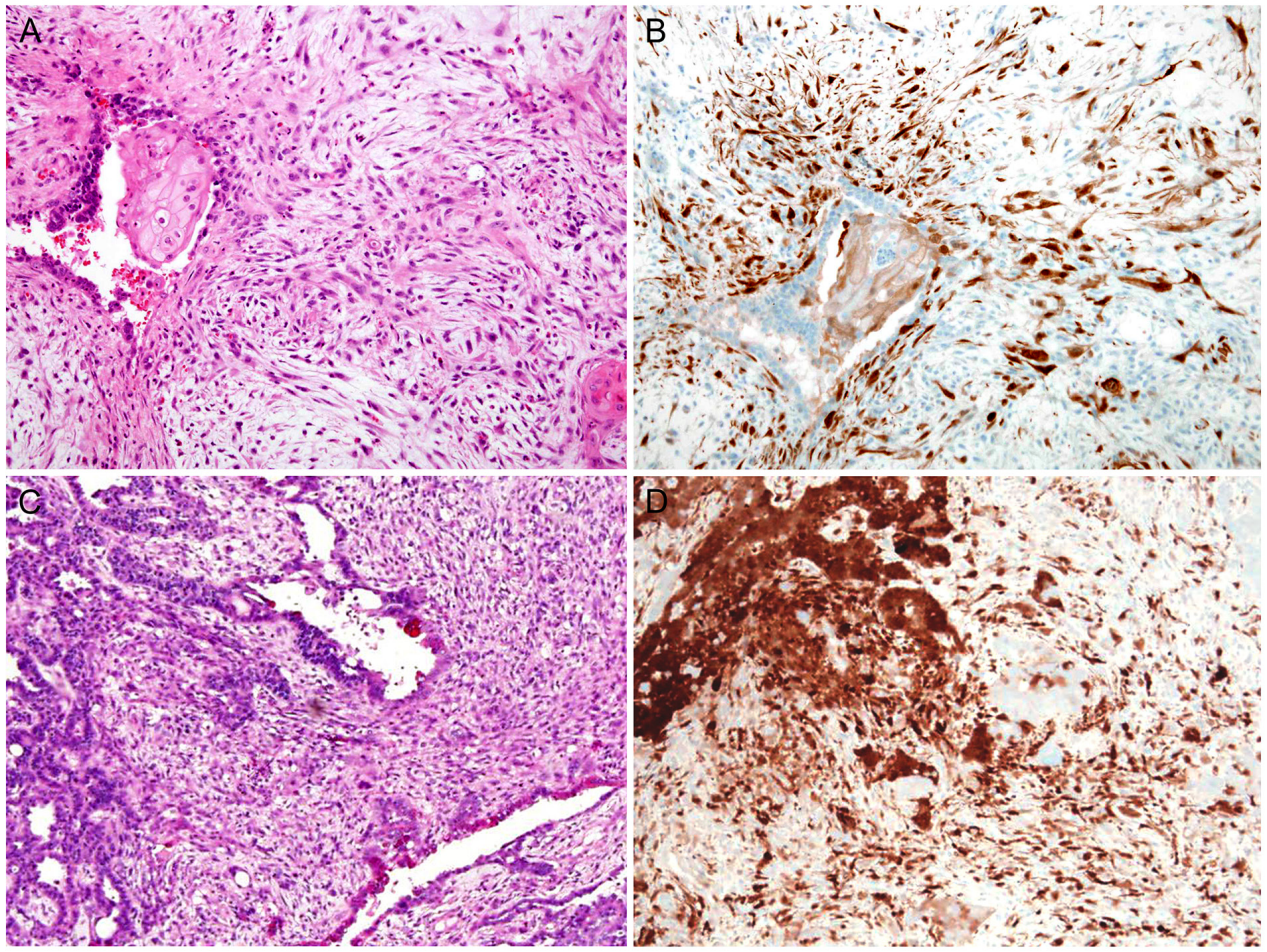
Stromal p16 overexpression in malignant endometrial lesions: Carcinosarcoma **A**. Carcinosarcoma showing EC with squamous differentiation as the epithelial component. **B**. The stromal componentexhibits diffuse, strong p16 immunoreactivity, whereas the epithelial component is negative for p16. **C**. Carcinosarcoma showing SC as the epithelial component. **D**. Both the tumor cells (left upper corner) and stroma exhibit diffuse, strong p16 immunoreactivity.

Representative photomicrographs of stromal p16 expression in malignant endometrial lesions are presented in Figure 3 (EC and SC) and Figure 4 (carcinosarcoma). Of the 36 cases of malignant endometrial lesions, 29 cases (80.6%) showed p16 immunostaining scores of ≥ 4. All of the remaining 7 cases with scores of ≤ 3 were EC; none of the cases of SC or carcinosarcoma displayed scores of ≤ 3. Moreover, 7 (87.5%) of the 8 SC cases and 6 (85.7%) of the 7 carcinosarcoma cases showed scores of ≥ 6. In contrast, in EC, 10 (47.6%) of the 21 cases had scores of ≥ 6. Stromal p16 expression levels were significantly higher in SC than in EC (*P* = 0.021), whereas the difference between carcinosarcoma and SC was not statistically significant (*P* = 0.483).

### Differences in stromal p16 expression between benign, precancerous, and malignant endometrial lesions

The median p16 immunostaining scores of benign, precancerous and malignant endometrial lesions were 0, 3.5, and 6, respectively. To analyze the differences in stromal p16 expression between groups classified according to the degree of malignancy, a linear-by-linear association test was performed (Table 2). A significant difference was observed in stromal p16 expression between the benign and precancerous lesions (*P* < 0.001). Moreover, stromal p16 expression differed significantly between the precancerous and malignant groups (*P* = 0.005).

**Table 2:** Differences in stromal p16 expression between benign, precancerous, and malignant endometrial lesions

Category	Total	p16 immunostaining score							*P* value
		0	1	2	3	4	6	9	
Benign	62	33 (53.2)	16 (25.8)	10 (16.1)	3 (4.8)	0 (0.0)	0 (0.0)	0 (0.0)	<0.001 (vs Precancerous);<0.001 (vs Malignant)
Precancerous	26	1 (3.8)	3 (11.5)	4 (15.4)	5 (19.2)	7 (26.9)	4 (15.4)	2 (7.7)	0.005 (vs Malignant)
Malignant	36	2 (5.6)	0 (0.0)	1 (2.8)	4 (11.1)	6 (16.7)	14 (38.9)	9 (25.0)	

We classified endometrial lesions into two clusters based on the histological type and degree of malignancy. The first cluster consisted of nonpathological endometrium, endometrial polyp, hyperplasia without atypia, AH/EIN, and EC (Table 3). Stromal p16 expression levels were not significantly different between nonpathological endometrium and endometrial polyp (*P* = 0.457). Similarly, stromal p16 expression levels in hyperplasia without atypia were not significantly different from nonpathological endometrium or endometrial polyp (*P* = 0.920). In contrast, AH/EIN exhibited significantly higher levels of stromal p16 expression compared with hyperplasia without atypia (*P* < 0.001). A significant difference was also noted in stromal p16 expression when comparing EC with AH/EIN (*P* = 0.012).

**Table 3:** Differences in stromal p16 expression between nonpathological endometrium, endometrial polyp, hyperplasia without atypia, AH/EIN, and EC

Category	Pathological diagnosis	Total	p16 immunostaining score							*P* value
			0	1	2	3	4	6	9	
Benign	Nonpathological endometrium	10	5 (50.0)	3 (50.0)	2 (50.0)	0 (0.0)	0 (0.0)	0 (0.0)	0 (0.0)	<0.001 (vs Precanceorus);<0.001 (vs Malignant)
	Endometrial polyp	17	8 (47.1)	3 (17.6)	4 (23.5)	2 (11.8)	0 (0.0)	0 (0.0)	0 (0.0)	
	Hyperplasia without atypia	14	5 (35.7)	7 (50.0)	1 (7.1)	1 (7.1)	0 (0.0)	0 (0.0)	0 (0.0)	
Precancerous	AH/EIN	21	1 (4.8)	3 (14.3)	4 (19.0)	5 (23.8)	6 (28.6)	2 (9.5)	0 (0.0)	0.012 (vs Malignant)
Malignant	Endometrioid carcinoma	21	2 (9.5)	0 (0.0)	1 (4.8)	4 (19.0)	4 (19.0)	7 (33.3)	3 (14.3)	

**Abbreviations:** AH/EIN: atypical hyperplasia/endometrioid intraepithelial neoplasia

The second cluster consisted of atrophic endometrium, endometrial polyp, SEIC, and SC (Table 4). SC generally arises in the setting of an atrophic, and not a hyperplastic, endometrium or an endometrial polyp [11]. No significant difference in stromal p16 expression was observed between atrophic endometrium and endometrial polyp, even though there was a trend toward increased expression in endometrial polyp (*P* = 0.088). SEIC exhibited significantly higher levels of stromal p16 expression compared with atrophic endometrium or endometrial polyp (*P* < 0.001). All cases of SEIC showed scores of ≥ 4, whereas all cases of atrophic endometrium and endometrial polyp showed scores of ≤ 3. In contrast with the endometrioid type, there was no statistically significant difference in stromal p16 expression between SEIC and SC (*P* = 0.788).

**Table 4:** Differences in stromal p16 expression between atrophic endometrium, endometrial polyp, SEIC, and SC

Category	Pathological diagnosis	Total	p16 immunostaining score							*P* value
			0	1	2	3	4	6	9	
Benign	Atrophic endometrium	13	9 (69.2)	3 (23.1)	1 (7.7)	0 (0.0)	0 (0.0)	0 (0.0)	0 (0.0)	<0.001 (vs Precancerous);<0.001 (vs Malignant)
	Endometrial polyp	17	8 (47.1)	3 (17.6)	4 (23.5)	2 (11.8)	0 (0.0)	0 (0.0)	0 (0.0)	
Precancerous	SEIC	5	0 (0.0)	0 (0.0)	0 (0.0)	0 (0.0)	1 (20.0)	2 (40.0)	2 (40.0)	0.788 (vs Malignant)
Malignant	Serous carcinoma	8	0 (0.0)	0 (0.0)	0 (0.0)	0 (0.0)	1 (12.5)	3 (37.5)	4 (50.0)	

**Abbreviations:** SC: serous carcinoma, SEIC: serous endometrial intraepithelial carcinoma

## DISCUSSION

A novel finding reported in this preliminary study is the gradual and significant increase in stromal p16 expression with increased degree of malignancy in benign, precancerous, and malignant endometrial lesions. We observed that more than 90% (59/62) of the benign lesions had p16 immunostaining scores of < 3, whereas the precancerous lesions showed scores of 3 to 6 in approximately 60% (16/26) of the cases examined. Moreover, more than half (23/36) of malignant lesions displayed scores of 6 or more. Consistent with this finding, a comparison of p16 expression within tumors of each histological type also revealed significant differences. In endometrioid neoplasms, the differences in stromal p16 expression between precancerous and benign lesions (*P* < 0.001) and between malignant and precancerous lesions (*P* = 0.012) were statistically significant. Also in serous neoplasms, stromal p16 expression was significantly higher in malignant and precancerous lesions than in benign lesions (*P* < 0.001). In addition, stromal p16 expression was found to have 100% sensitivity and 100% specificity when the cutoff value is p16 immunostaining score 4 (Table 5). These findings are consistent with those of our previous study demonstrating stromal p16 overexpression in ovarian carcinoma [21]. Compared with benign and borderline ovarian neoplasms, ovarian carcinomas had significantly elevated p16 expression levels in the peritumoral stroma. These significances remained when the analysis was performed based on lesion classification as serous, mucinous, and endometriosis-associated neoplasms. Our observation of significantly higher levels of stromal p16 expression in malignant endometrial lesions suggests that p16 may be involved in tumor cell growth and invasion in the tumor microenvironment through its overexpression in stromal cells. Some previous studies have reported p16 overexpression at the invasive tumor front of endometrial carcinoma, colorectal carcinoma, and basal cell carcinoma [22–25]. These results suggest that p16 may be involved in tumor invasion and progression and support the hypothesis that the p16 protein promotes invasiveness through interactions with other molecules related with tumor cell migration and invasion [6, 22–24, 26]. To confirm our preliminary results, it will be necessary to analyze stromal p16 expression using a larger number of endometrial carcinoma samples.

**Table 5:** Sensitivity, specificity, positive predictive value, and negative predictive value of stromal p16 expression in endometrial lesions

Category		Sensitivity	Specificity	PositivePredictive value	NegativePredictive value
Benign vs Precancerous/Malignant		75.61%	100.0%	100.0%	67.74%
Endometrioid	Benign vsPrecancerous/Malignant	68.33%	100.0%	100.0%	53.66%
Serous	Benign vs Precancerous/Malignant	100.0%	100.0%	100.0%	100.0%

We demonstrated that stromal p16 expression was significantly higher in endometrial SC than in endometrial EC. Seven of the 8 (87.5%) of SC cases showed p16 immunostaining scores of ≥ 6, whereas 52.4% (11/21) of the EC cases had scores of ≤ 4. The majority of endometrial SC displayed diffuse, strong p16 expression. In addition to p53 protein, which typically shows aberrant expression pattern in SC, p16 is also used as a diagnostic biomarker for differentiating EC from SC. The characteristic immunohistochemical findings of endometrial SC, such as diffuse, strong p16 expression and aberrant p53 expression, are observed in the tumor cells. However, there has been no report about the expression pattern of p16 and p53 proteins in the peritumoral stroma of SC. Our observation suggests that the significantly higher p16 expression levels in SC compared with EC may reflect the more aggressive oncogenic behavior, such as invasiveness, metastatic ability, and recurrence, and worse prognosis of SC compared with those of EC. Consistent with this finding, SEIC showed significantly higher stromal p16 expression levels than AH/EIN. SEIC, a putative precursor of SC, can be associated with intra-abdominal carcinoma. In other words, even in the absence of definitive invasion in the uterus, SEIC can behave like SC and result in peritoneal carcinomatosis. Our observation suggests that the significantly higher p16 expression levels in SEIC compared with AH/EIN may reflect the more aggressive nature of SEIC compared with that of AH/EIN.

We also demonstrated that stromal p16 expression levels are significantly higher in carcinosarcoma than in EC, but not in SC. Histopathologically, EC corresponds to type I endometrial carcinoma, whereas SC is the prototype of type II endometrial carcinoma. Carcinosarcoma is also considered as a type II endometrial carcinoma. Type I endometrial carcinoma develops from normal endometrial glandular epithelium under the influence of estrogenic stimulation, through a continuum of histopathologically recognizable endometrial hyperplasia. The overall survival rate for patients with type I endometrial carcinoma exceeds 80%. In contrast, type II endometrial carcinoma is known to be unrelated to estrogenic stimulation and have an aggressive behavior. Such lesions are characterized by deep myometrial invasion, frequent nodal and extrauterine metastases, and poor outcome; approximately 50% of patients with type II endometrial carcinoma develop recurrences [27]. A significant difference in stromal p16 expression levels between type I and II endometrial carcinomas raises the possibility that the aggressive oncogenic behavior of type II lesions may be associated with p16 overexpression in the peritumoral stroma.

We found one previous study reporting stromal p16 expression in endometrial neoplastic lesions [28]. For 80.0% (28/35) of endometrial polyp cases, p16 immunoreactivity with moderate or greater intensity was observed in fibrous stroma, and 1 (3.0%; 1/33) case of endometrial hyperplasia showed weak p16 expression; however, none of the endometrial carcinoma cases (0.0%; 0/23) showed stromal p16 expression. Moritani et al. [28] stated that stromal p16 expression was a characteristic finding of endometrial polyps and was useful in differentiating between endometrial hyperplasia and endometrial polyps. These results are inconsistent with our findings that stromal p16 expression is significantly higher in precancerous and malignant lesions than in benign lesions. We observed that all endometrial polyp cases examined had p16 immunostaining score of ≤ 3; 47.1% (8/17) of the endometrial polyp cases showed no stromal p16 expression. Moreover, 64.3% (9/14) of the endometrial hyperplasia cases had weak or greater expression. The difference in stromal p16 expression between endometrial polyp and hyperplasia without atypia was not statistically significant. On the basis of our data, one cannot differentiate between endometrial polyp and hyperplasia without atypia using stromal p16 expression status. In addition, both EC and AH/EIN exhibited significantly higher stromal p16 expression levels compared with hyperplasia without atypia or endometrial polyp. This result was opposite to that of the previous study by Moritani et al. [28]. We attribute these differences to the following two reasons. First, two different sets of tissue samples were obtained from Japanese and Korean patients, and stromal p16 expression patterns may be race-specific. Second, p16 overexpression was observed in benign tumors such as benign nevus, neurofibroma, and schwannoma, which are related to oncogene-induced cellular senescence [28]. Thus, p16 overexpression in benign lesions inhibits cellular proliferation, protecting cells from malignant transformation [6]. The significantly higher rates of stromal p16 overexpression in endometrial polyps can be explained by oncogene-induced cellular senescence. In contrast, in this study, precancerous and malignant lesions showed higher levels of stromal p16 expression, which might be due to a positive feedback mechanism caused by RB protein deregulation.

Another study on p16 expression in the stroma of ductal carcinoma *in situ* (DCIS) of the breast [29] provided evidence that DCIS with high stromal p16 expression tended to show estrogen receptor negativity and high Ki-67 labeling indices. In addition, it was reported that high stromal p16 expression was a strong independent predictor of DCIS recurrence with a higher hazard ratio than the established prognostic markers. These findings are in agreement with our data. p16 is an inhibitor of cell growth in response to various stress stimuli, such as DNA damage, oxidative stress, or hyperproliferative signals. Therefore, p16 protein induces cellular senescence, such that stromal p16 overexpression is indicative of stromal cell senescence. On the basis of the results of previous studies, [28, 30, 31], we postulated that senescent stroma can contribute to disease progression by secreting inflammatory mediators, cytokines, and enzymes such as proteases, providing a mechanism through which p16-positive stroma contributes to tumor progression and/or invasion.

In conclusion, we investigated the stromal p16 expression status in endometrial lesions by immunohistochemical staining. We found that stromal p16 expression of malignant endometrial lesions was significantly higher than that of precancerous lesions, which in turn was significantly higher than that of benign endometrial lesions. Stromal p16 expression was absent or weak in benign lesions, whereas the majority of malignant lesions exhibited diffuse and moderate-to-strong p16 immunoreactivity, suggesting that stromal p16 expression promotes the development and progression of endometrial carcinoma. Further studies are necessary to confirm our preliminary results.

## MATERIALS AND METHODS

### Tissue specimens

Between March 2015 and May 2016, 124 cases of endometrial lesions were retrieved from the surgical pathology files of Severance Hospital. The pathological diagnoses are summarized in Table 1. The endometrial lesions were classified as benign, precancerous, and malignant in 62, 26, and 36 cases, respectively. Of the 62 cases of benign lesions, 15 (24.2%), 10 (16.1%), 2 (3.2%), and 35 (56.5%) cases were diagnosed during dilatation and curettage (D&C), hysteroscopic resection, total abdominal hysterectomy (TAH), and total laparoscopic hysterectomy (TLH), respectively. Of the 26 cases of precancerous lesions, 7 (26.9%), 2 (7.7%), 2 (7.7%), 4 (15.4%), and 11 (42.3%) cases were diagnosed during D&C, hysteroscopic resection, robot-assisted total hysterectomy, TAH, and TLH, respectively. Of the 36 cases of malignant lesions, 2 (5.6%), 2 (5.6%), 12 (33.3), and 20 (55.6%) cases were diagnosed during radical abdominal hysterectomy, radical laparoscopic hysterectomy, TAH, and TLH, respectively. This study did not include any cases where the histological differential diagnosis between benign and precancerous lesions was ambiguous. None of the patients received preoperative neoadjuvant chemotherapy, radiation therapy, or concurrent chemoradiation therapy. Pathological diagnoses were classified following the criteria of the WHO Classification of Tumours of Female Reproductive Organs, revised in 2014 [11]. In particular, in the revised 2014 WHO Classification, endometrial hyperplasia was classified into hyperplasia without atypia and AH/EIN on the basis of the presence of cytological atypia. This study was reviewed and approved by the Institutional Review Board at the Severance Hospital, Yonsei University Health System, Seoul, Republic of Korea (2016-1371-001).

### Histopathological examination

The curetted or resected specimens were fixed in 10% neutral-buffered formalin and embedded in paraffin blocks. From each formalin-fixed, paraffin-embedded block, 4-μm sections were cut and stained with hematoxylin and eosin. Two independent pathologists examined all available hematoxylin and eosin-stained slides by routine light microscopy and chose the most representative slide to perform immunohistochemical staining.

### Immunohistochemical staining

The formalin-fixed, paraffin-embedded sections were deparaffinized and rehydrated with a xylene and alcohol solution. Immunohistochemical staining was performed using a Ventana Benchmark XT automated staining system (Ventana Medical Systems, Tucson, AZ, USA), according to the manufacturer's instructions. Antigen retrieval was performed using Cell Conditioning Solution (CC1; Ventana Medical Systems). Sections were incubated with primary antibodies against p16 (pre-diluted, clone E6H4, Ventana Medical Systems). After chromogenic visualization, using UltraView Universal DAB Detection Kits (Ventana Medical Systems), slides were counterstained with hematoxylin. Appropriate positive and negative controls were concurrently stained to validate the staining method.

The percentage of p16-positive stromal cells and the staining intensity were assessed. The cut-off index was defined as the presence of 10% or more cells displaying nuclear p16 immunoreactivity, as described previously [21, 28, 32]. The estimated percentages were categorized as follows: less than 10% (score 0), 10% to 24% (score 1), 25% to 50% (score 2), or 50% or more (score 3). The staining intensity was graded as follows: negative (score 0), weak (score 1), moderate (score 2), or strong (score 3). The subcellular location of p16-positive signals (nuclear or cytoplasmic) was also estimated. The final score was calculated as the product of the percentage and staining intensity, resulting in scores of 0, 1, 2, 3, 4, 6, and 9 [33].

### Statistical analysis

Linear-by-linear association test was performed to compare the status of stromal p16 expression between histological types and to determine whether stromal p16 expression was significantly different according to the degree of malignancy. Statistical analyses were performed using PASW Statistics 18 (IBM SPSS, Chicago, IL, USA). Statistical significance was set at *P* < 0.05.
